# Cerebellar Neurocysticercosis as Long-Term Complication of Allogeneic Haematopoietic Stem Cell Transplantation from Haploidentical Donor

**DOI:** 10.1155/2019/4603130

**Published:** 2019-09-19

**Authors:** Federico Meconi, Giulia Ciangola, Benedetta Mariotti, Raffaella Cerretti, Laura Cudillo, William Arcese, Alessandra Picardi

**Affiliations:** University Tor Vergata, Department of Biomedicine and Prevention, Rome, Italy

## Abstract

Neurocysticercosis, an infection of the central nervous system with the larval stage of the cestode *Taenia solium*, is uncommon in developed countries. We report a case of allogeneic haematopoietic stem cell transplantation from a haploidentical donor complicated, in the long term, by *T. solium* infection of the central nervous system and successfully treated with empiric antiparasitic therapy with albendazole plus dexamethasone. Revised diagnostic criteria proposed by Del Brutto et al. were used for the definitive diagnosis of cerebellar neurocysticercosis.

## 1. Introduction

Cysticercosis is the most common parasitic disease of the central nervous system (CNS) in humans [1], caused by infection of *Taenia solium*, the larval stage of the tapeworm found in pigs. Human cysticercosis results from fecal-oral contamination with *Taenia solium* eggs from a human tapeworm carrier. People living in rural setting are at increased risk for exposure. Clinical manifestations are nonspecific and depend on locations, number, size of cysts, and host immune response to the cysticerci. In the CNS, it frequently involves the cerebral hemispheres and cortex and rarely involves ventricles and basal cisterns, subarachnoid space, and spine [2]. When the cysts coalesce to form a tree-like pattern it is known as racemose neurocysticercosis (NCC). Diagnosis of NCC is often difficult due to dangerous brain localization and lack of laboratory specific tests. Revised diagnostic criteria, proposed by Del Brutto et al. [3], as reported in Table 1, may be useful for the definitive diagnosis of NCC.

Here in, we report a case of a cerebellar racemose NCC as a long-term complication of allogeneic haematopoietic stem cell transplantation (HSCT) from a haploidentical donor.

## 2. Case Presentation

A 64-year-old female underwent haploidentical allogeneic HSCT for diagnosis of acute myeloid leukemia secondary to myelodysplastic syndrome in September 2016. A haploidentical son, 41 years old, 0 Rh positive, EBV, CMV, and toxoplasma IgG positive, was selected as the donor because of the lack of a well-matched unrelated stem cell donor according to our transplant policy [4, 5].

The Valencia schedule, consisting of a combination of reduced dose of thiotepa, Busilvex, and fludarabine, was used [6] as a conditioning regimen, while the association of cyclosporine (0.75 mg/Kg/12 h continuous infusion from day –7, increased from day –1 to 1.5 mg/Kg/12 h continuous infusion), a short course of methotrexate (15 mg/mq on day 1 and 10 mg/mq on day 3–6 and 11), rabbit anti-thymocyte globulin (ATG) (5 mg/Kg/die from day –4 to day –1), basiliximab (20 mg on day 0 and day +4), and mycophenolic acid (15 mg/Kg twice daily) was administered as GVHD prophylaxis.

On day +100 after transplant, she developed acute gastrointestinal (GI) tract (stage 1, grade 2) GVHD successfully treated with steroids. Transplant-related complications due to the consequent use of a high-dose steroid for long time for GVHD included multiple episodes of reactivation of cytomegalovirus responded to antiviral therapy, sarcopenia, and fracture of the head of the femur surgically resolved.

On day +320 after transplant, the patient presented with nausea, vomiting, nystagmus, diplopia, and severe cutaneous purpura. A CT scan of the head showed a right multicystic cerebellar mass with perilesional edema compressing the fourth ventricle, also confirmed by brain MRI. As the lesions were not previously reported, we ruled out congenital abnormalities such as cavernous malformations. Since other differential diagnosis, including infectious (tuberculotic, echinococcal, and toxoplasmotic cysts) and neoplastic diseases, required a histological assessment, we proceeded to a surgical excision of one of the neuroradiological lesions.

**Figure 1:**
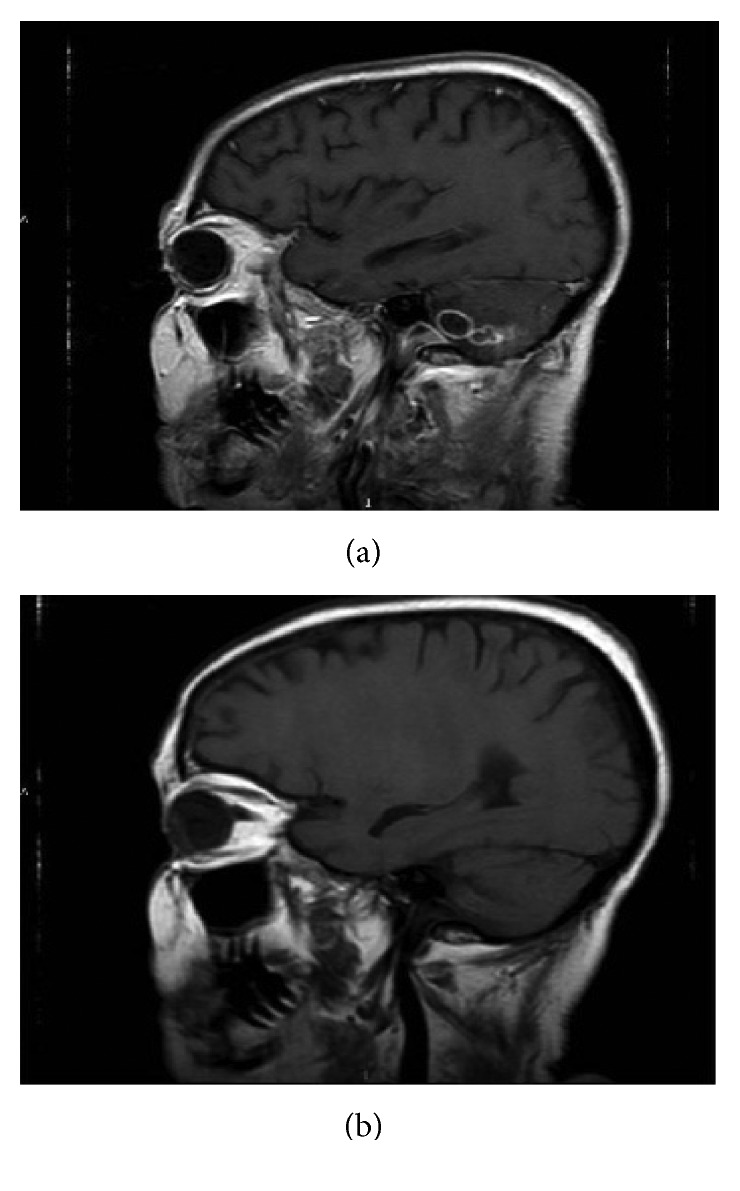
(a) MRI before starting albendazole. (b) MRI after 10 days of albendazole.

An attempted biopsy of the cystic lesions, through suboccipital craniotomy, failed due to moderate bleeding during surgery. Histologic findings showed normal cerebellar tissue with polymorphonuclear leukocytes. Although the available specimen was not sufficient for all diagnostic tests, no neoplastic cells or fungal hyphae were seen. The fundus oculi assessment did not show cystis. Moreover, the quantiferon and TB skin test (or PPD test) were negative. Although prompt empiric antibiotic combination of meropenem and clindamycin was started, clinical and neuroradiological response was not achieved. High-dose intravenous trimethoprim-sulfamethoxazole for a suspicious cerebral toxoplasmosis, due to donor and patient IgG positivity before HSCT, was given after 20 days of antibiotic therapy. After two weeks of this therapy, continued clinical symptoms and MRI of the brain showed worsening of multicystic lesions with perifocal edema and initial cerebellar herniation. The patient also developed an intention tremor, bilateral hypoacusia, and severe haematological toxicity. In absence of clinical and radiological improvement and taking into account the patient's history of living in countryside, we applied the revised diagnostic criteria for *Taenia solium* infection and discovered that our patient presented a definitive diagnosis of NCC due to the neuroimaging presence of cystic lesions without a discernible scolex and enhancing lesion plus previous household contact with *T. solium* and neurological clinical manifestations of NCC. Moreover, in this case we documented the resolution of cystic lesions after cysticidal drug therapy (Figure 1), as confirmative criterion.

However, no cystic lesions were found in muscles, and the IgG Elisa test in blood and 3 consecutive stool samples were negative for *T. Solium*. The patient started empiric antiparasitic treatment with albendazole (400 mg PO BID) plus intravenous administration of dexamethasone (4 mg/day). Lumbar puncture was not performed due to high risk of hernia wedging and MRI signs of hydrocephalus. After 14 days, brain MRI control showed disappearance of some of the cystic lesions in the right cerebellar hemisphere and the reduction in size of some others. Patient was released from the hospital with no neurological symptoms, continuing albendazole therapy for four months.

## 3. Discussion

Despite the fact that NCC is the most common parasitic infection of the CNS and one of the main causes of acquired epilepsy worldwide [7], the diagnosis process is still difficult and sometimes it is feasible only through the exclusion of similar diseases. Clinical symptoms are very heterogeneous, depending on the location of cystic lesions in the CNS. Acquired seizures are the most common symptoms but also chronic headaches, focal neurological deficits, intracranial hypertension, and cognitive decline are described as frequent. Furthermore, it is not always possible to biopsy these lesions to obtain histological confirmation of the parasite colonization due to localization in the CSN. According to Del Brutto et al. revised diagnostic criteria, in the case reported here, the definitive diagnosis was made thanks to the association of neuroimaging with clinical features that together may be considered highly sensitive and specific of this rare disease as well described by Bustos et al. [8].

In developed countries, CNS infection by uncommon parasites is very rare, also in patients with severe immunosuppression due to chemotherapy, HSCT, and HIV. However, emerging germs must be reconsidered due to the migration flux from other countries where they are still endogenous.

Since immunosuppression is deeper in the haematopoietic stem cell transplants than organ transplants, patients' anamnestic history should include questions about previous contact with parasites. Moreover, specific hygienic rules should be recommended in discharged patients. Finally, living in rural areas or contact with swines must be strongly advised against after the HSCT procedure.

To the best of our knowledge, there is no evidence that previous exposure to *T. Solium* is an absolute contraindication to HSCT. Furthermore, it is still matter of debate if these patients could benefit of a prophylaxis or a pre-emptive antiparasitic therapy as it is now recommended for *P. jirovecii*, cytomegalovirus, and herpes viruses.

Barra Valencia et al. have reported one case of CNS cystercosis infection after transplant for solid organs [9]; only two reports of NCC have been reported as short-term complication (3–4 months) after allogeneic haematopoietic stem cell transplant (HSCT) [10, 11]. This case report represents a rare cerebellar racemose cysticercosis localization of *T. Solium* as long-term complication of haploidentical HSCT and underlines the adjunctive risk of the environmental factors, beyond the slow immunological recovery, on the transplant outcome of this subset of patients.

This case report highlights a rare and dangerous case of an opportunistic infection, presenting as a late complication after haploidentical allogeneic transplant and the importance of collecting complete patient's history before transplant in order to identify all individual risk factors. Reestablishment of immunocompetence requires at least several months, and some patients continue to demonstrate immune deficits for several years after HSCT (in general NK cells are the first lymphocyte subset to recover, followed by CD8+ T cells, B cells, and ultimately CD4+ T cells). CD4+ count is probably the most predictive marker of the restoration of immune competence, and several studies have demonstrated that CD4+ recovery is associated with diminished infectious risk and improved transplant outcomes. Nowadays, there is a clinical need for specific environmental guidelines for this cohort of patients to avoid behaviors at high infectious risk. Safe living after HSCT includes preventing infections primarily transmitted by direct contact (hand hygiene), infections primarily transmitted through respiratory exposures (contact with persons with respiratory illnesses or plants, soil, or their aerosols), and pet-transmitted zoonotic infections. Additional food safety practices appropriate for HSCT recipients include avoiding undercooked meat and using separated cutting boards [9]. Moreover, in this setting, the possibility and timing for resuming previous habits in life should be better defined. Finally, the recent introduction of chimeric antigen receptor T cells [10] infusions represents a very promising tool to improve the immunological recovery after haploidentical HSCT and could minimize the development of CNS infections too.

## Figures and Tables

**Table 1:** Revised diagnostic criteria by Del Brutto et al. [3].

Categories of criteria	Criteria
*Absolute*	(i) Histological demonstration of the parasite from biopsy of a brain or spinal cord lesion
	(ii) Visualization of subretinal cysticercus
	(iii) Conclusive demonstration of a scolex within a cystic lesion on neuroimaging studies

*Neuroimaging*	*Major neuroimaging criteria*
	(i) Cystic lesions without a discernible scolex
	(ii) Enhancing lesions^a^
	(iii) Multilobulated cystic lesions in the subarachnoid space
	(iv) Typical parenchymal brain calcifications^a^
	*Confirmative neuroimaging criteria*
	(i) Resolution of cystic lesions after cysticidal drug therapy
	(ii) Spontaneous resolution of single small enhancing lesions^b^
	(iii) Migration of ventricular cysts documented on sequential neuroimaging studies^a^
	*Minor neuroimaging criteria*
	(i) Obstructive hydrocephalus (symmetric or asymmetric) or abnormal enhancement of basal leptomeninges

*Clinical/exposure*	*Major clinical/exposure*
	(i) Detection of specific anticysticercal antibodies or cysticercal antigens by well-standardized immunodiagnostic tests^a^
	(ii) Cysticercosis outside the central nervous system^a^
	(iii) Evidence of a household contact with *T. solium* infection
	*Minor clinical/exposure*
	(i) Clinical manifestations suggestive of neurocysticercosis^a^
	(ii) Individuals coming from or living in an area where cysticercosis is endemic^a^
	*Definitive diagnosis*
	(i) One absolute criterion
	(ii) Two major neuroimaging criteria plus any clinical/exposure criteria
	(iii) One major and one confirmative neuroimaging criteria plus any clinical/exposure criteria
	(iv) One major neuroimaging criteria plus two clinical/exposure criteria (including at least one major clinical/exposure criterion) together with the exclusion of other pathologies producing similar neuroimaging findings
	*Probable diagnosis*
	(i) One major neuroimaging criteria plus any two clinical/exposure criteria
	(ii) One minor neuroimaging criteria plus at least one major clinical/exposure criteria

^a^Operational definitions. Cystic lesions: rounded, well-defined lesions with liquid contents of signal similar to that of the CSF on CT or MRI; enhancing lesions: single or multiple, ring- or nodular-enhancing lesions of 10–20 mm in diameter, with or without surrounding edema, but not displacing midline structures; typical parenchymal brain calcifications: single or multiple, solid, and most usually <10 mm in diameter; migration of the ventricular cyst: demonstration of a different location of ventricular cystic lesions on sequential CTs or MRIs; well-standardized immunodiagnostic tests: so far, antibody detection by the enzyme-linked immunoelectrotransfer blot assay using lentil lectin-purified *T. solium* antigens, and detection of cysticercal antigens by monoclonal antibody-based ELISA; cysticercosis outside the central nervous system: demonstration of cysticerci from biopsy of subcutaneous nodules, X-ray films, or CT showing cigar-shaped calcifications in soft tissues, or visualization of the parasite in the anterior chamber of the eye; suggestive clinical manifestations: mainly seizures (often starting in individuals aged 20–49 years; the diagnosis of seizures in this context is not excluded if patients are outside of the typical age range), but other manifestations include chronic headaches, focal neurologic deficits, intracranial hypertension, and cognitive decline; cysticercosis-endemic area: a place where active transmission is documented. ^b^The use of corticosteroids makes this criterion invalid.
